# Flavonolignans As a Novel Class of Sodium Pump Inhibitors

**DOI:** 10.3389/fphys.2016.00115

**Published:** 2016-03-30

**Authors:** Martin Kubala, Petra Čechová, Jaroslava Geletičová, Michal Biler, Tereza Štenclová, Patrick Trouillas, David Biedermann

**Affiliations:** ^1^Department of Biophysics, Faculty of Science, Centre of Region Haná for Biotechnological and Agricultural Research, Palacký UniversityOlomouc, Czech Republic; ^2^INSERM UMR 850, School of Pharmacy, University LimogesLimoges, France; ^3^Department of Physical Chemistry, Faculty of Science, Regional Centre of Advanced Technologies and Materials, Palacký UniversityOlomouc, Czech Republic; ^4^Laboratory of Biotransformation, Institute of Microbiology, Czech Academy of SciencesPrague, Czech Republic

**Keywords:** sodium pump, Na^+^/K^+^-ATPase, flavonolignans, inhibition, binding sites

## Abstract

We examined the inhibitory effects of three flavonolignans and their dehydro- derivatives, taxifolin and quercetin on the activity of the Na^+^/K^+^-ATPase (NKA). The flavonolignans silychristin, dehydrosilychristin and dehydrosilydianin inhibited NKA with IC_50_ of 110 ± 40 μM, 38 ± 8 μM, and 36 ± 14 μM, respectively. Using the methods of molecular modeling, we identified several possible binding sites for these species on NKA and proposed the possible mechanisms of inhibition. The binding to the extracellular- or cytoplasmic C-terminal sites can block the transport of cations through the plasma membrane, while the binding on the interface of cytoplasmic domains can inhibit the enzyme allosterically. Fluorescence spectroscopy experiments confirmed the interaction of these three species with the large cytoplasmic segment connecting transmembrane helices 4 and 5 (C45). The flavonolignans are distinct from the cardiac glycosides that are currently used in NKA treatment. Because their binding sites are different, the mechanism of inhibition is different as well as the range of active concentrations, one can expect that these new NKA inhibitors would exhibit also a different biomedical actions than cardiac glycosides.

## Introduction

Sodium pump (Na^+^/K^+^-ATPase, E.C. 3.6.3.9, NKA) is an enzyme of crucial importance for all animal cells. It is the major determinant of cytoplasmic Na^+^ and K^+^ concentrations and the resting plasma membrane potential. The steep Na^+^ gradient on plasma membrane is essential for variety of secondary active transporters, e.g., Na^+^/Ca^2+^- and Na^+^/H^+^- exchanger or Na^+^-dependent glucose transporter, and hence, NKA indirectly regulates also concentrations of other physiologically important solutes.

It is not surprising that an uncontrolled inhibition of NKA can result in severe diseases, e.g., renal failure, hypertension or diabetic neuropathies (Kaplan, 2002) or even death, and that the most specific NKA inhibitor cardiac glycoside ouabain was originally used as an arrow poison (Newman et al., 2008). Despite these risks, extracts containing cardiac glycosides were used to control heart tonics already in ancient medicine, and the extracts were prepared either from plants in Arabic medicine (Brewer, 2004) or secretions of frog *Bufo bufo* in Chinese medicine (Watabe et al., 1996). Compounds like digitalis or digoxin are still prescribed for control of congestive heart failure (Gheorghiade et al., 2004). However, the use of cardiac glycosides is limited by their very narrow useful concentration range (Newman et al., 2008), which stimulates further search for other NKA inhibiting compounds.

**Figure 1:**
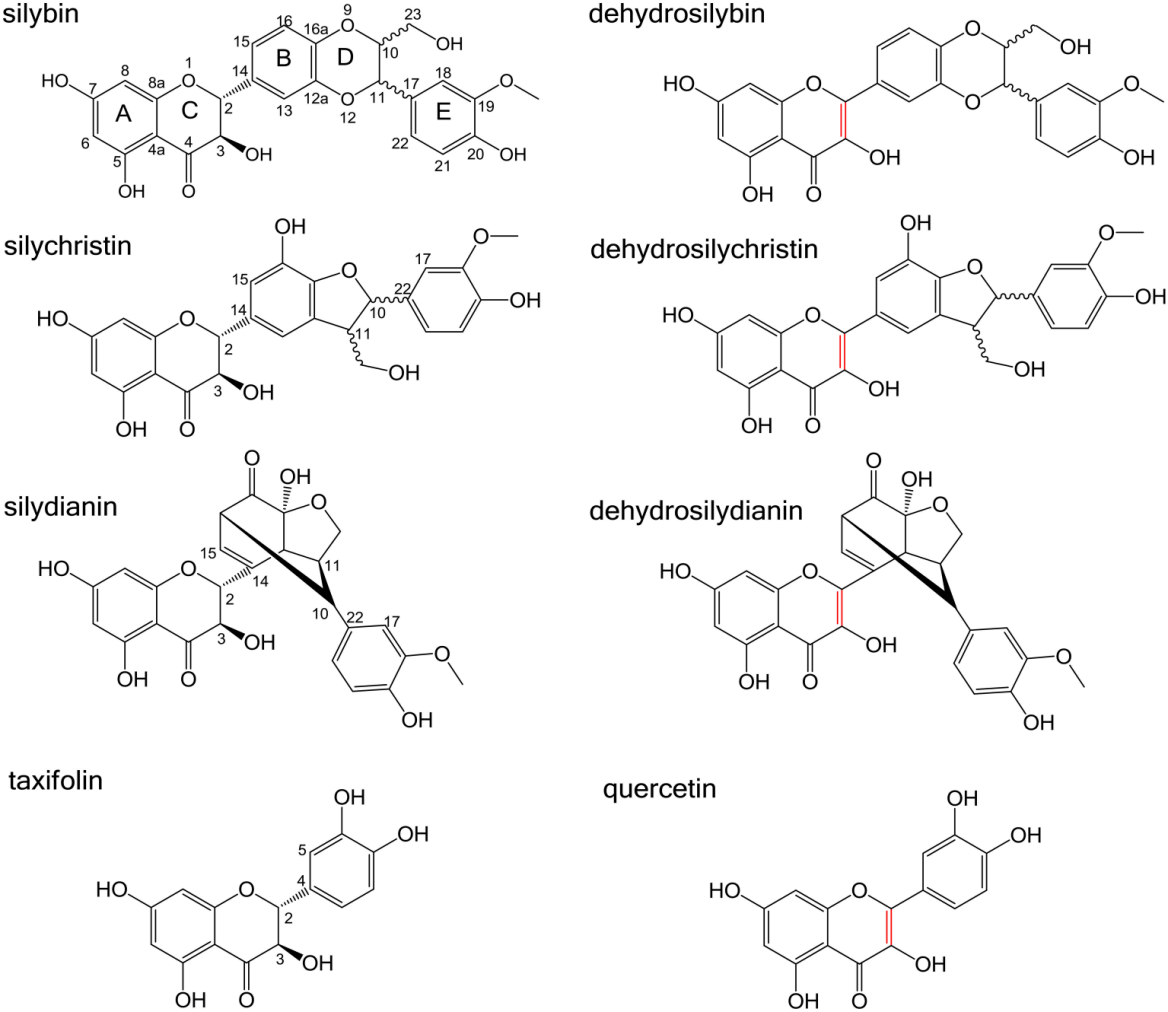
**Structures of silybin (SB), silychristin (SCH), silydianin (SD) and their dehydro-derivatives (DHSB, DHSCH, DHSD), taxifolin (TAX), and quercetin (QUE)**. The 2,3-double bond in dehydroderivatives is highlighted in red.

Silymarin is an extract from the seeds of milk thistle (*Silybum marianum)*. It contains numerous polyphenolic compounds and it was shown to possess antioxidant (Vacek et al., 2013; Pyszková et al., 2015), hepatoprotective (Loguercio and Festi, 2011) or anticancer effects (Agarwal et al., 2006). In this study, we have tested effects on NKA activity for a flavonoid taxifolin (TAX) and three flavonolignans, namely silybin (SB), silychristin (SCH), and silydianin (SD), which are major silymarin compounds (Biedermann et al., 2014); their structures are shown in Figure 1. The corresponding 2,3-dehydro derivatives (DHSB, DHSCH, and DHSD, the 2,3-dehydrotaxifolin is termed quercetin, QUE) were also tested.

The NKA catalytic cycle is usually described by the Albers-Post cycle (Jorgensen et al., 2003). It postulates that during the catalytic cycle, the enzyme adopts two major conformations, denoted as E1 and E2. In E1, the enzyme has high affinity to sodium and ATP and the binding sites are open to the cytoplasm, while in E2, the enzyme has high affinity to potassium, low affinity to ATP and the cation-binding sites are open to the extracellular space. High-resolution structures of NKA were obtained in several conformational states thanks to recent progresses in X-ray crystallography of membrane proteins (Morth et al., 2007; Ogawa et al., 2009; Nyblom et al., 2013). They revealed the binding sites for transported cations within the transmembrane domain, or binding site for some ligands, including ouabain. Notably, in the crystals assigned to the enzyme in E1 conformation, the cytoplasmic domains are assembled together (further referred to as a closed conformation), while in the E2 conformation, the cytoplasmic headpiece is widely opened (opened conformation).

These different structures provided a solid basis to figure out molecular processes responsible for the mechanisms of enzyme inhibition. Moreover, based on these high-resolution structures, molecular modeling allows further identification of the binding sites of flavonolignans that inhibit NKA, and strongly support rationalization of mechanisms of inhibition.

## Materials and methods

### Chemicals

Unless stated otherwise, all used chemicals were from Sigma-Aldrich Chemie (Steinheim, Germany).

### Species tested for the effect on NKA activity

Silymarin was purchased from Liaoning Senrong Pharmaceutical Co., Ltd. (China), SB, SCH, and SD were isolated from the silymarin, and the dehydro- derivatives DHSB, DHSCH and DHSD were prepared as described previously (Džubák et al., 2006; Křenek et al., 2014; Pyszková et al., 2015). Silybin and silychristin were used as the natural diastereomeric mixture (ca. 1/1 and 95/5 respectively), silydianin is a single isomer. Dehydroderivatives were prepared from parent mixtures and are therefore enantiomeric mixtures (besides DHSD) (Pyszková et al., 2015). Taxifolin was purchased from Amagro (CZ). Quercetin was prepared by acidic hydrolysis of rutin with HCl/EtOH as described previously (Wang et al., 2011). Purity of all used compounds was over 95% (HPLC, PDA).

### Isolation of Na^+^/K^+^-ATPase

**Figure 2:**
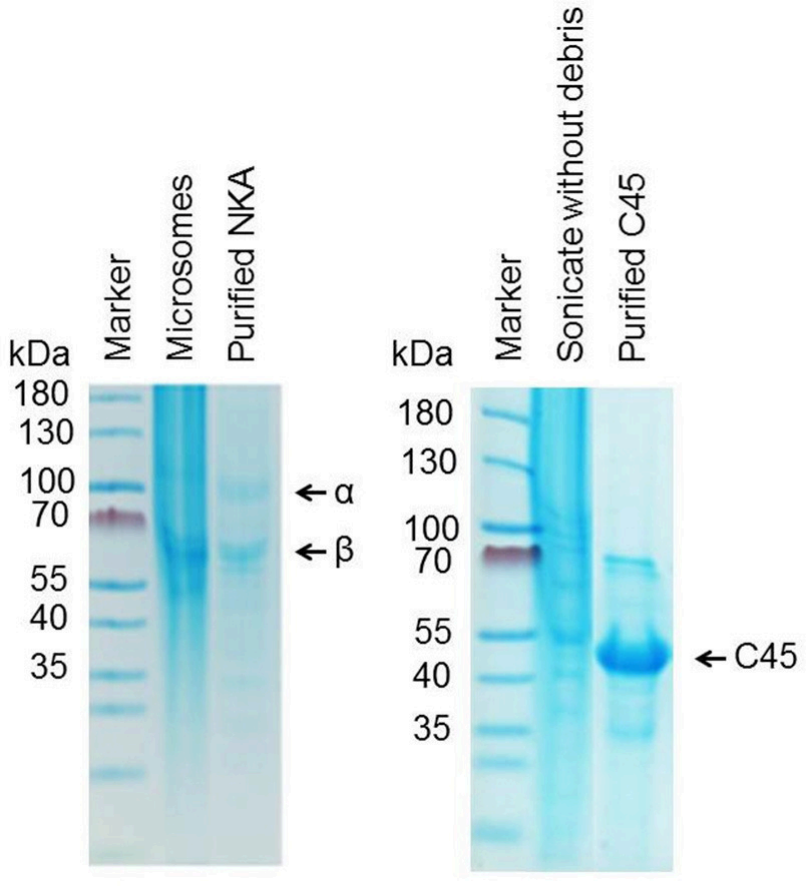
**Purity of isolated NKA (left) and C45 (right) as revealed by SDS-PAGE**.

The NKA was prepared from porcine kidney outer medulla using the method of Jorgensen and Klodos with some modifications (Jørgensen, 1988; Klodos et al., 2002; Kubala et al., 2014). An isolated enzyme was pipetted into small aliquots and stored at −20°C in ISE buffer (25 mM imidazole, 250 mM sucrose, 1 mM EDTA, pH 7.4) containing SDS detergent. The molar concentration of isolated NKA was estimated using the Bradford method with consideration of MW(α + β) = 165,000 Da. The protein purity >90% was estimated from SDS-PAGE (Figure 2).

### Measurement of ATPase activity

The measurements of NKA activity were performed using the Baginsky assay (Kubala et al., 2014). The assay was performed in microwell plates in 4 replicates for each point and automated in the pipetting station Freedom EVO (Tecan, Switzerland).

The reaction buffer was composed of 325 mM NaCl, 50 mM KCl, 7.5 mM MgCl_2_, and 75 mM imidazole, pH 7.2. In the experiments where the K^+^-dependence was estimated, the KCl concentration is ranging from 0 to 100 mM. The NKA (0.1 mg/mL) isolated from porcine kidney was mixed with reaction buffer without ATP. All inhibitors were solubilized in methanol immediately before the measurement and then premixed with the reaction buffer to the required concentration. Subsequently, 10 μL of inhibitor solution was added into 20 μL of enzyme solution and incubated for 2 min. In the control sample, only 10 μL of reaction buffer without inhibitor was added including corresponding amount of methanol. The reaction was started by the addition of ATP solution (20 μL, 7.5 mM in the stock, the final concentration in the reaction was 3 mM). The reaction proceeded for 6 min at room temperature and then was stopped by addition of 75 μL of staining solution, which was composed of 160 mM ascorbic acid, 3.7% (v/v) acetic acid, 3% (w/v) SDS, and 0.5% ammonium molybdate. The staining reaction was stopped after another 8 min by adding 125 μL of solution composed of 0.9% (w/v) bismuth citrate, 0.9% (w/v) sodium citrate and 3.7% HCl.

The Baginsky method detects a product of ATP hydrolysis, inorganic phosphate, which interacts with ammonium molybdate. The reaction results in a color change, which can be monitored as a change of absorbance at 710 nm, and was measured using microplate reader Synergy Mx (BioTek, USA). The calibration line was determined using KH_2_PO_4_ solutions, in 0–37.5 nM concentration range.

The specific NKA activity is standardly estimated using the treatment by ouabain, which serves as a specific inhibitor of NKA. The ATPase activity decreases to less than 10% in the presence of 10 mM ouabain. This residual activity in the presence of ouabain was subtracted from the total estimated ATPase activity in ouabain-untreated samples, and all data are presented as the ouabain-sensitive ATPase activity. The IC_50_ values were obtained from fitting the data to the logistic function.

### Expression and purification of the isolated large cytoplasmic segment C45

The large cytoplasmic segment connecting the transmembrane helices 4 and 5 (C45 loop, residues L354-I777 of the mouse brain sequence) with a (His)_6_-tag at the N-terminus was expressed in *E. coli* BL21 (Promega, USA) and purified using a Co^2+^-based affinity resin (Clontech, USA) as described previously (Grycova et al., 2009). Immediately after elution, the protein was dialyzed into 20 mM Tris, 140 mM NaCl, pH 7.4 buffer and stored at −20°C.

Protein were analyzed by Coomassie blue stained SDS PAGE and concentration was determined using the Bradford assay (Bradford, 1976) using BSA as a standard.

### Absorption and fluorescence spectroscopy

Spectroscopic experiments were performed in 20 mM Tris, 140 mM NaCl, pH 7.4 buffer. SCH, DHSCH and DHSD as well as protein C45 were diluted to 5 μM concentration.

Absorption spectra were measured on spectrometer Specord 250 Plus (Analytic Jena, Germany) in the range 300–600 nm, with the bandpass 2 nm, a step 1 nm and a scan-speed 2 nm/s. The reference spectrum was acquired using the cuvette with a pure buffer.

Fluorescence emission spectra were measured using Fluorolog-3 (Horiba Scientific, USA). The spectra were scanned with a step of 1 nm, both excitation- and emission bandpass 5 nm and integration time 1 s. For SCH, the excitation was 325 nm and emission was recorded in the 340–600 nm interval, for DHSCH and DHSD, the excitation wavelength was 380 nm and spectra were scanned in the 400–600 nm interval. Signal from pure buffer was subtracted as a background.

### Computation of structures for molecular docking

Geometry optimizations of all structures (Figure 1) was performed using the density functional theory (DFT) formalism with the software package Gaussian09 (Frisch et al., 2009). The hybrid functional B3P86 has been used because it has repeatedly succeeded in describing most of polyphenol properties (Trouillas et al., 2006, 2008). Gibbs energies (G) were computed at B3P86/6-31+G(d,p) level at 298 K, 1 atm. After a vibrational frequency analysis, ground-state geometries were confirmed by the absence of any imaginary frequency. Quantum calculations were performed in the gas phase.

The SB (stereoisomer A) and DHSB (stereoisomer A) initial structures were taken from Trouillas et al. (2008) and further re-optimized. Structure of the most stable isomers of SCH (stereoisomer A), DHSCH (stereoisomer A), SD and DHSD were already presented in Pyszková et al. (2015).

### Molecular docking

The compounds SCH, DHSCH, and DHSD were docked to the opened and closed structures of NKA (PDB, ID, 3KDP, and 4HQJ) using Autodock Tools (Morris et al., 2009) and Autodock Vina (Trott and Olson, 2010) with the grid covering the whole protein. The values of parameters exhaustiveness was set to 100 and num_modes to 9999 in order to reveal all possible docking modes. In the default setting, the bonds creating different conformers were freely rotable, to find optimal geometry of molecules interacting with the pump.

## Results

### Conformational analysis

Conformational analysis of both flavonoids TAX and its dehydro- analog QUE revealed two stable rotamers. In case of QUE, as reported previously (Trouillas et al., 2006), the planarity is observed, allowing π electron delocalization along the A, C and B rings, while for TAX, the planarity is lost due to the loss of the 2,3-double bond.

**Figure 3:**
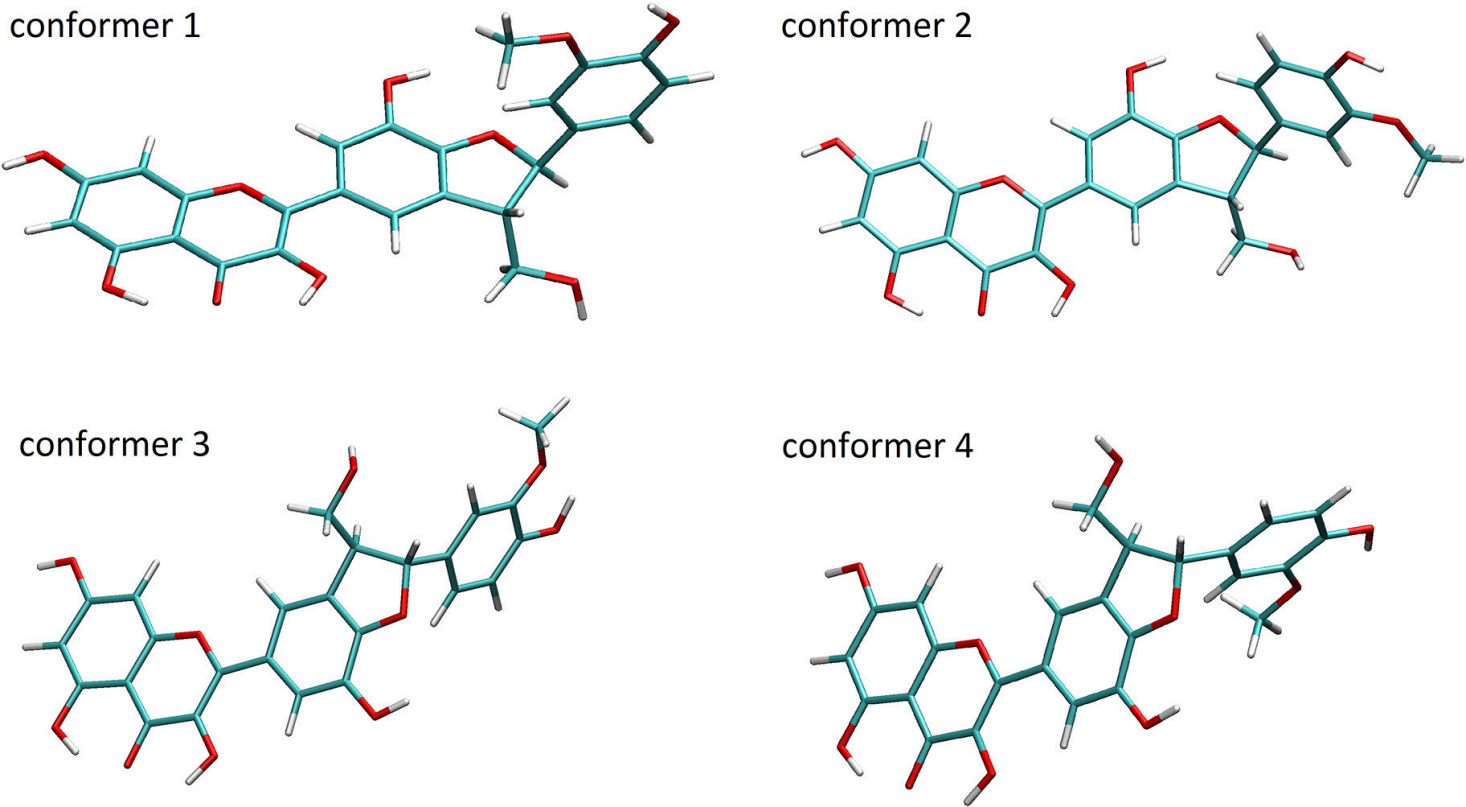
**Structures of stable conformers of DHSCH**.

A conformation analysis of SB, DHSB, SCH, DHSCH, SD, and DHSD revealed 4 most stable representative conformers for all molecules within less than 1.6 kcal/mol difference in Gibbs energy (Figure 3). These conformers can be obtained by modification of the torsion angles Phi = C3–C2–C14–C15 and Psi = C11–C10–C22–C17 (Psi = C10–C11–C17–C22 for SB and DHSB) (Table 1), and the loss of the flavonoid moiety planarity is observed also for these molecules, when the 2,3-bond is hydrogenated (DHSB, DHSCH, and DHSD).

**Table 1:** **Structural parameters of the stable conformers**.

**Species**	**Conformer**	**Phi (°)**	**Psi (°)**	**Relative Gibbs energy (kcal/mol)**
SB	1	77	−98	0
	2	83	81	0.4
	3	−96	−97	0.2
	4	−98	81	0.2
DHSB	1	3	−98	0
	2	−1	80	0.3
	3	−178	−98	0.1
	4	−176	83	0.5
SCH	1	81	−71	0
	2	82	98	0.9
	3	−105	52	0.1
	4	−97	−141	0.7
DHSCH	1	174	−52	0
	2	175	87	0.2
	3	9	97	0.1
	4	7	−55	1.6
SD	1	−3	−37	0
	2	−5	147	0.2
	3	−125	−42	0.5
	4	−123	142	0.4
DHSD	1	1	−45	0
	2	1	142	0.3
	3	166	−40	0.2
	4	166	145	0.8
TAX	1	−83		0
	2	80		0.1
QUE	1	180		0
	2	0		0.4

### Effects on NKA activity

**Figure 4:**
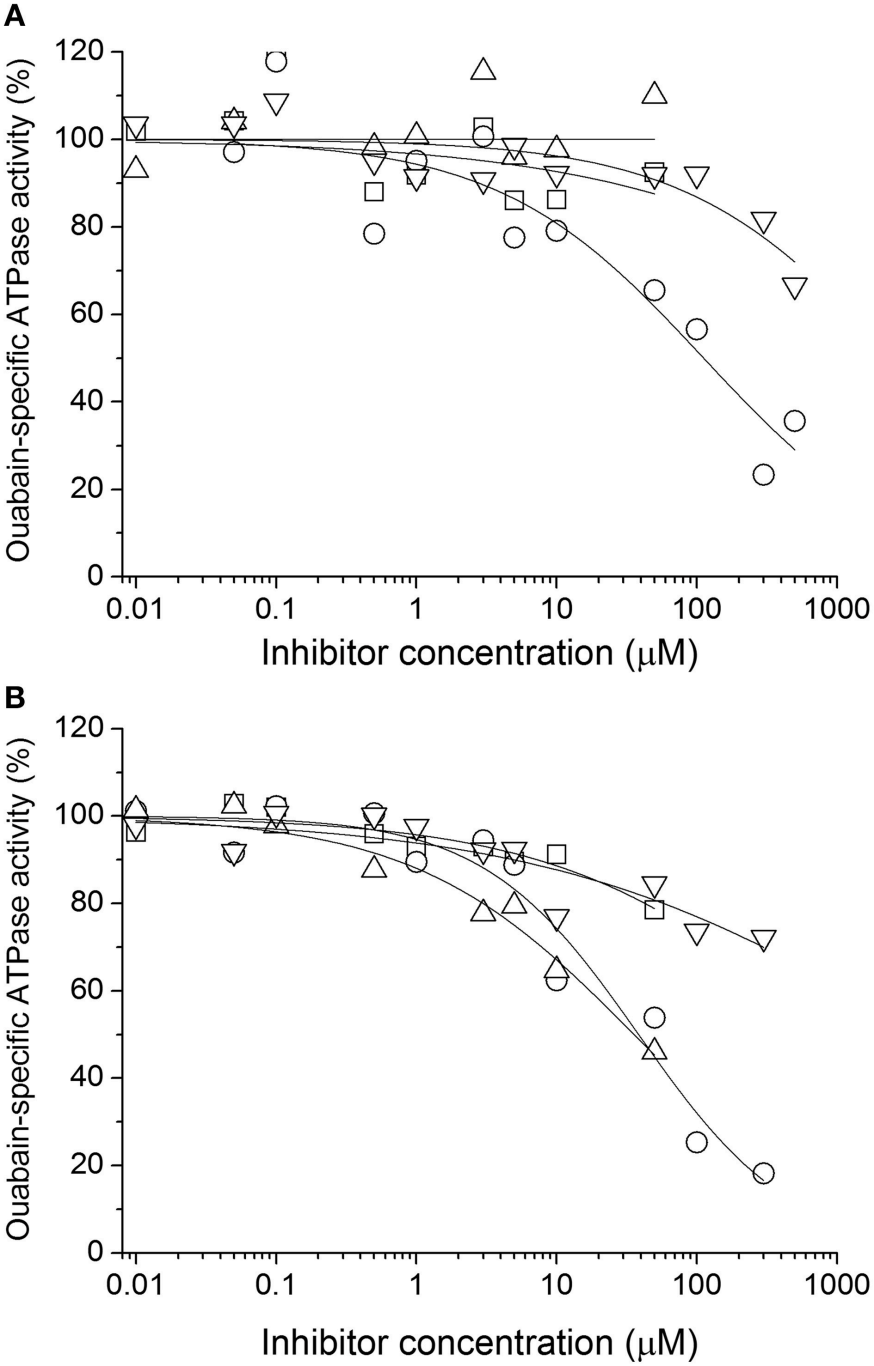
**Inhibition of NKA by (A) SB (squares), SCH (circles), SD (up triangles), TAX (down triangles) and (B) by the corresponding dehydro- derivatives DHSB, DHSCH, DHSD and QUE**. The reference value (100%) represents ouabain-specific activity of untreated NKA.

Ouabain-sensitive ATPase activity was measured for increasing concentrations of all species (Figure 4). In the case of SB, DHSB, SD, and DHSD, the examined concentration range was limited due to lower solubility of these species. Substantial inhibition was observed only for SCH, DHSCH and DHSD with IC_50_ 110 ± 40 μM, 38 ± 8 μM, and 36 ± 14 μM, respectively. In all these cases, we observed a Hill coefficient < 1, indicating the presence of multiple binding sites and negative cooperativity. The data are summarized in Table 2, and SCH, DHSCH, and DHSD were subject to further analyses.

**Table 2:** **Values of IC_50_ for inhibition of the NKA activity, n, Hill coefficient, K_0.5_(ouabain) indicates the value obtained in the presence of 5 mM KCl (in parentheses in 40 mM KCl) and 40 μM concentration of flavonolignan, K_0.5_(K^+^) denotes the values for K^+^-dependent activation of NKA obtained in the presence of 40 μM flavonolignan, n.d., not determined**.

**Species**	**IC_50_ (μM)**	**n**	**K_0.5_(ouabain) (μM)**	**K_0.5_(K^+^) (mM)**
None			2.6 ± 1.3 (23 ± 3)	4.2 ± 0.6
SB	>1000	n.d.	n.d.	n.d.
DHSB	900 ± 800	0.5 ± 0.1	n.d.	n.d.
SCH	110 ± 40	0.6 ± 0.1	4.0 ± 1.7 (18 ± 2)	8 ± 5
DHSCH	38 ± 8	0.8 ± 0.1	1.5 ± 0.5 (24 ± 6)	4 ± 1
SD	>1000	n.d.	n.d.	n.d.
DHSD	36 ± 14	0.6 ± 0.1	3.3 ± 1.4 (19 ± 7)	3 ± 2
TAX	>1000	n.d.	n.d.	n.d.
QUE	>1000	n.d.	n.d.	n.d.

**Figure 5:**
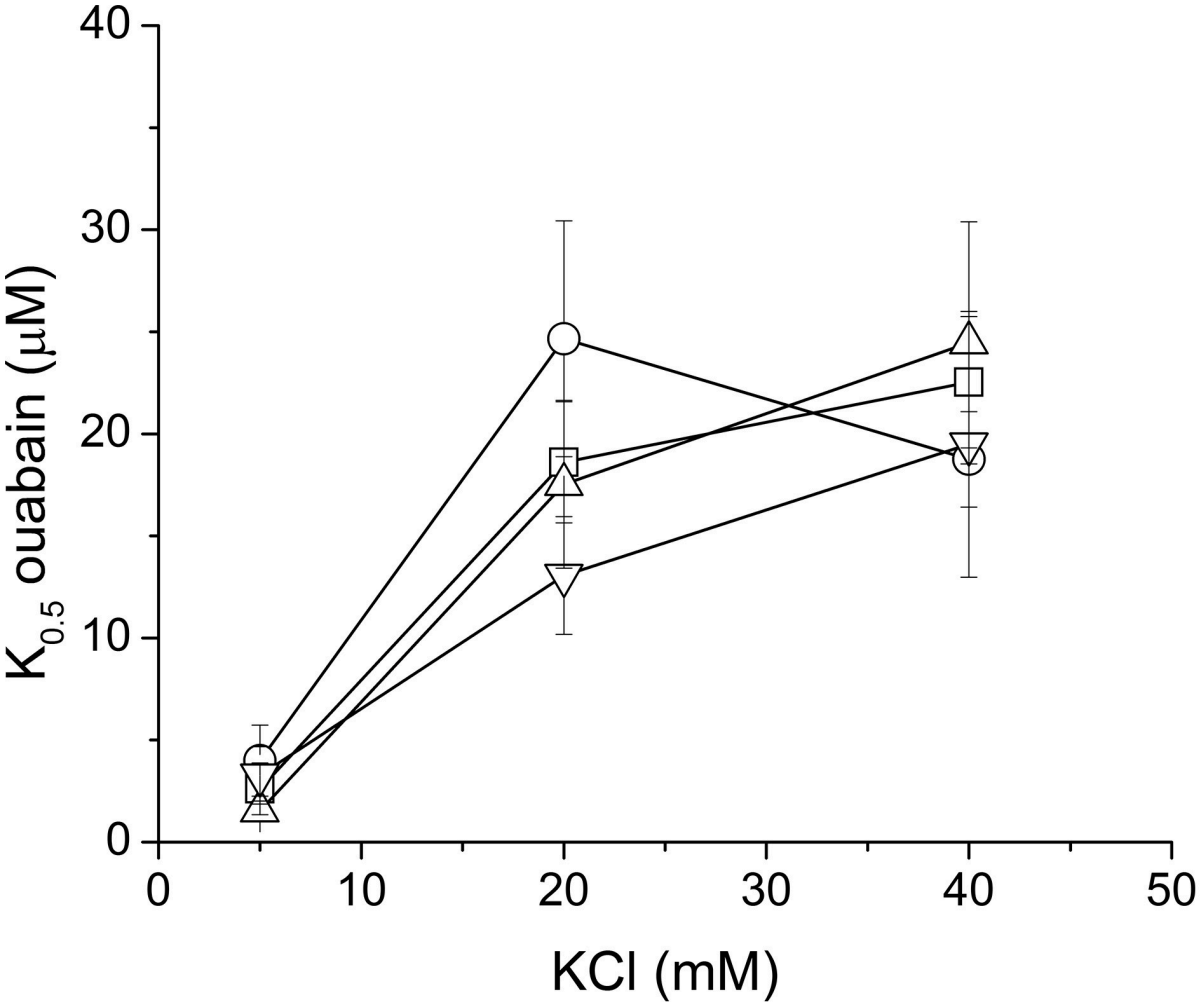
**The K^+^/ouabain antagonism in the absence (squares) or in the presence of 40 μM SCH (circles), DHSCH (up triangles) or DHSD (down triangles)**.

**Figure 6:**
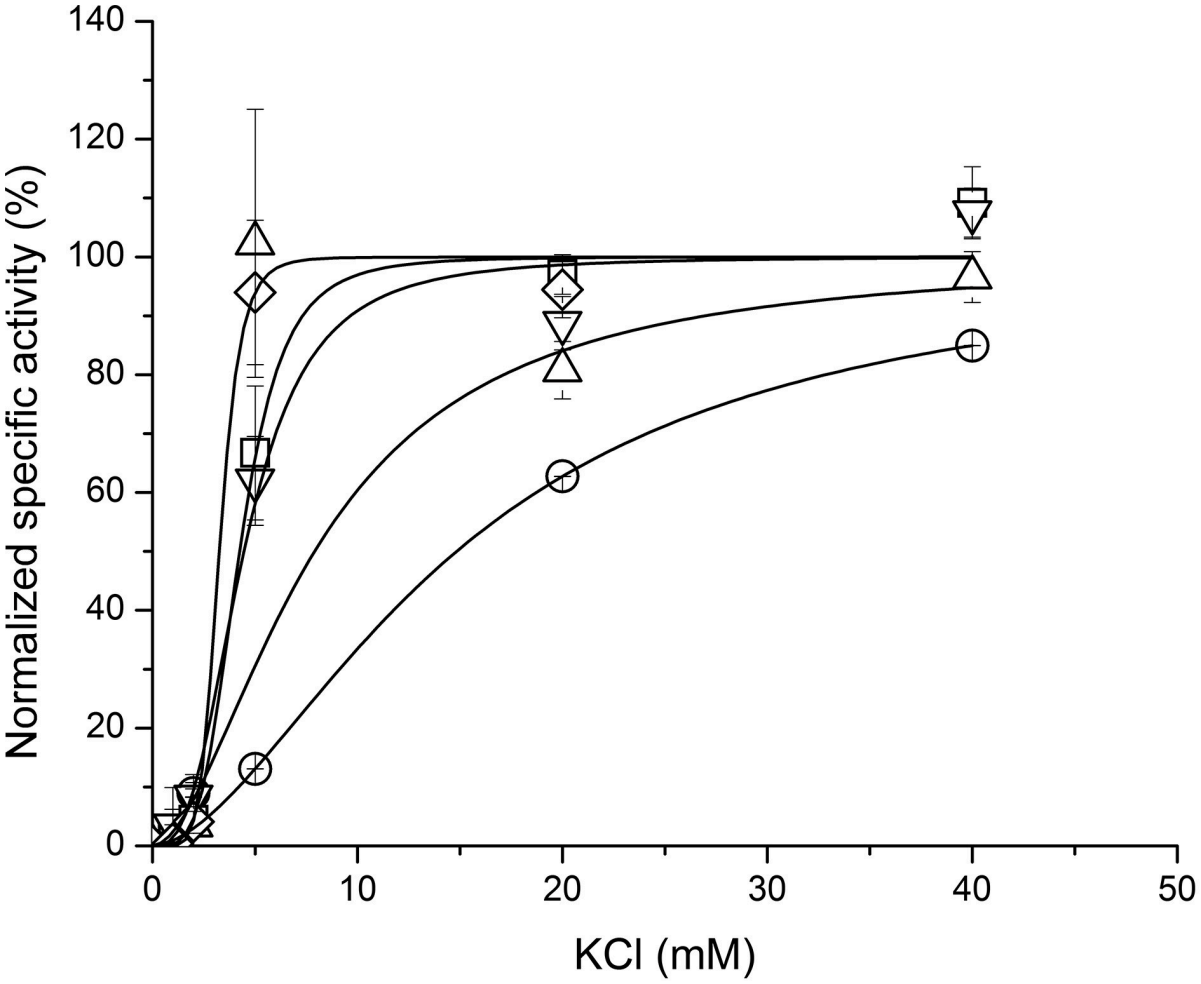
**The K^+^-dependence of NKA activity in the absence of any ligand (squares) or in the presence of ouabain (circles), SCH (up triangles), DHSCH (down triangles) or DHSD (diamonds)**.

We have tested influence of these three species on the ouabain inhibition and K^+^-dependence of NKA activation. None of the species substantially influenced the IC_50_ for ouabain or the K^+^/ouabain antagonism (Figure 5). The NKA activity is K^+^-dependent and it increased with increasing concentration of potassium with K_0.5_(K^+^) = 4.2 ± 0.6 mM. In contrast to ouabain, which raised the K_0.5_(K^+^) to 14.8 ± 0.1 mM, none of the flavonolignans significantly altered the K^+^-dependence of NKA activity (Figure 6).

### Molecular docking

**Figure 7:**
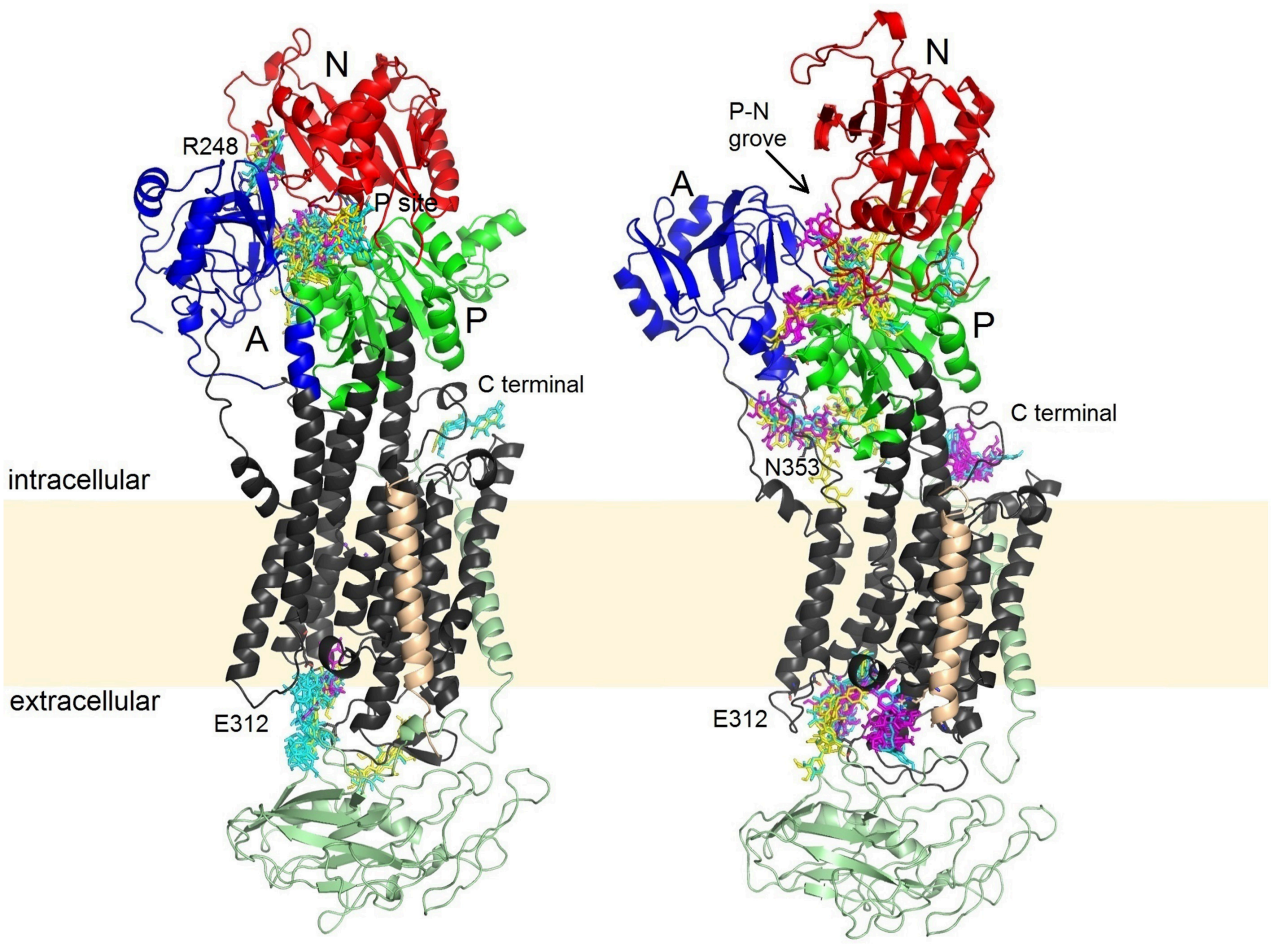
**Binding sites of SCH (yellow), DHSCH (cyan) and DHSD (magenta) on the opened and closed structure**. Beta subunit is in light green, the FXYD protein light orange, A, domain blue; N, domain red; P, domain green.

Molecular docking enables prediction of the binding sites for small ligands on large biomolecules. Binding to NKA was examined for both major conformations of the enzyme. All SCH, DHSCH, and DHSD bound with a similar affinities (−11 to −9 kcal/mol) to all sites in both opened and closed conformations. We identified five major binding sites, three of them were common to both the opened and closed conformations (Figure 7), one binding pose was exclusively observed only for the opened conformation, and another one in turn only for closed conformation. All conformers bound to at least one binding site, nevertheless, their relative preferences for individual binding sites differed (Table 3).

**Table 3:** **The fractions of conformers bound to the selected binding sites**.

**CLOSED**	**P site**	**R248**	**Extracellular**	**C terminal**	**Other**
DHSD1	**0.23**	0.05	0.03	ND	ND
DHSD2	**0.15**	ND	ND	ND	ND
DHSD3	**0.25**	ND	0.03	ND	ND
DHSD4	**0.28**	ND	ND	ND	ND
DHSCH1	**0.28**	ND	ND	0.03	0.03
DHSCH2	**0.23**	0.03	0.08	ND	0.05
DHSCH3	**0.13**	ND	0.03	ND	ND
DHSCH4	ND	0.03	**0.05**	0.03	0.05
SCH1	**0.15**	ND	0.03	0.03	0.05
SCH2	**0.33**	ND	ND	ND	ND
SCH3	**0.18**	0.03	0.03	ND	0.03
SCH4	**0.15**	ND	0.03	ND	ND
**OPENED**	**P site**	**N353**	**Extracellular**	**C terminal**	**Other**
DHSD1	**0.05**	0.03	0.03	0.03	0.05
DHSD2	0.03	**0.05**	ND	0.03	ND
DHSD3	0.05	ND	0.03	**0.10**	0.05
DHSD4	0.08	0.08	**0.15**	ND	0.20
DHSCH1	**0.10**	0.03	0.05	0.03	0.10
DHSCH2	0.05	**0.13**	ND	0.03	0.20
DHSCH3	ND	ND	ND	**0.03**	0.05
DHSCH4	0.08	**0.08**	0.03	ND	0.05
SCH1	**0.10**	0.05	0.05	ND	0.15
SCH2	**0.10**	ND	ND	ND	0.10
SCH3	**0.08**	0.05	0.05	ND	0.03
SCH4	**0.13**	0.05	ND	ND	0.08

The most preferred binding site of each conformer in bold, “P site” stands for phosphorylation site. ND, binding to this site was not detected.

**Figure 8:**
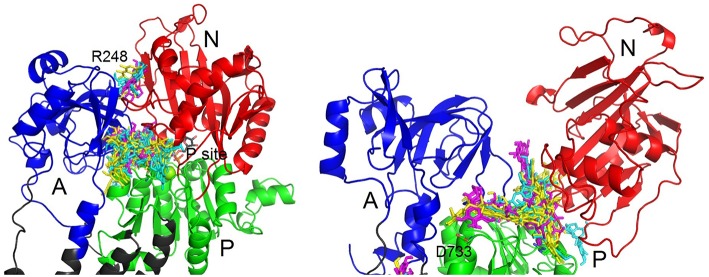
**Zoom on the binding sites of SCH (yellow), DHSCH (cyan) and DHSD (magenta) on cytoplasmic loops in the opened and closed structure**. A, domain is blue; N, domain red; P, domain green.

In the closed conformation, by far the most favored site was near the phosphorylation site. All the conformers can occupy this site, except for conformer 4 of DHSCH. All compounds bound also to another pose between the A and N domains on the other side near R248 (Figure 8). However, this site only exists in the closed conformation. It is much more selective and can be occupied only by conformer 3 of SCH, conformers 2 and 4 of DHSCH and conformer 1 of DHSD. Another possible binding location in the closed structure is on the extracellular side between E312 on TM4 and R886 (extracellular loop connecting TM7 and TM8), and it is the most favored site for conformer 4 of DHSCH. This site can serve as the entry for potassium ions during transport. Conformer 1 of SCH and conformers 1 and 4 of DHSCH are the only molecules that bind at the intracellular C-terminal site. Also this site was proposed to play a role in transport of cations through the plasma membrane (Toustrup-Jensen et al., 2009).

Also in the opened conformation, the binding to the groove between P and N cytoplasmic domains near the phosphorylation site was observed for all conformers, except for conformer 3 of DHSCH. It was the most preferred site for all SCH conformers and for conformers 1 of DHSCH and DHSD. The binding site specific only for the opened conformation is located under A domain, near the residues N353 and D740, where a regulatory potassium ion is bound (Schack et al., 2008). It is the most preferred site for conformers 2 and 4 of DHSCH and for conformer 2 of DHSD, but numerous other conformers can bind in this site as well. The site at the extracellular potassium entrance is the most preferred for the conformer 4 of DHSD. There is also a binding pose at the C-terminal of the protein, but it can be occupied only by DHSCH or DHSD, and for conformer 3 of both species it is the most preferred site.

### Interaction with the isolated large cytoplasmic segment C45

Based on the prediction from molecular docking, we examined whether SCH, DHSCH, and DHSD could interact with the isolated large cytoplasmic segment C45 using absorption and fluorescence spectroscopy. Absorption spectra of SCH, DHSCH, and DHSD exhibited maxima at ca. 325, 385, and 375 nm, respectively, and the presence of C45 did not substantially alter the spectra (not shown), thus, providing no further clue about the interaction.

**Figure 9:**
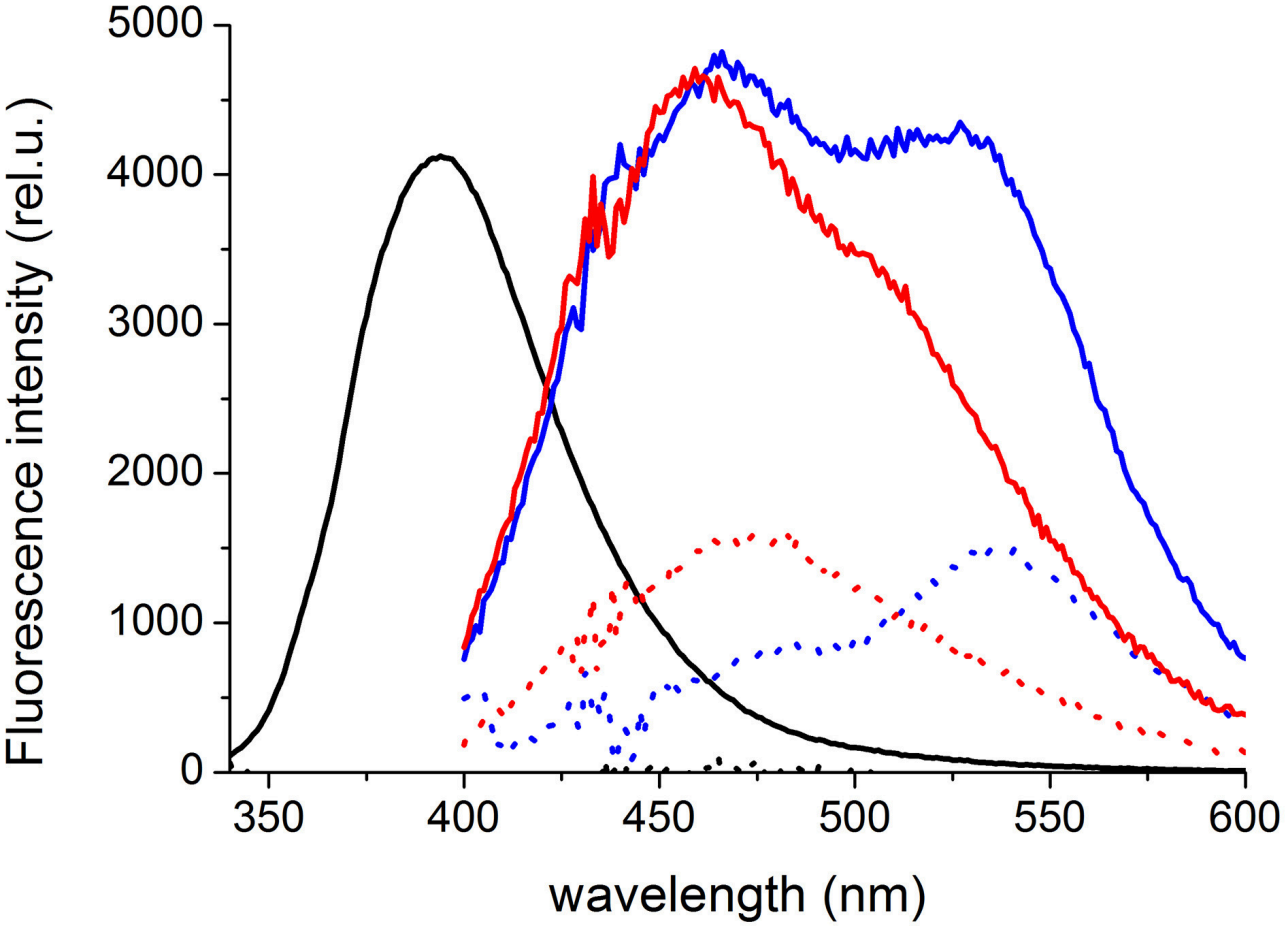
**Fluorescence emission spectra of 5 μM SCH (black), DHSCH (blue) and DHSD (red) in the presence (solid line) or absence (dotted line) of 5 μM C45**. The fluorescence intensity in the spectrum of SCH in the C45 presence was divided by a factor of 20 to fit the graph.

On the other hand, the interaction with C45 was clearly manifested in the fluorescence spectra for all three species. We observed no detectable fluorescence above background for the free SCH. The interaction with C45 protein turned out to be fluorogenic, and in the presence of C45, a bright SCH fluorescence appeared (quantum yield increased more than 1000 times compared to the free SCH) with a maximum at 394 nm (Figure 9). For the free DHSCH, we observed only very weak fluorescence with a maximum at 537 nm and an apparent shoulder at ca. 475 nm. In the presence of C45, the fluorescence intensity increased ca. 3-times, and two peaks at 466 and 527 nm could be resolved. Similarly for the free DHSD, there is only a weak fluorescence with a maximum at 472 nm. In the presence of C45, the three-fold intensity increase is accompanied by a shift of the maximum to 459 nm, and there is also an apparent shoulder at 510 nm.

## Discussion

Recent pharmacology greatly benefits from the species isolated from the plants used in traditional medicine. In addition, these species also serve as precursors or inspiration for synthesis of new active derivatives, which can turn out to be even more effective in biochemical interactions. This study was focused on interactions of a series of phenolic compounds from silymarin (being or not hydrogenated on 2,3-bond of the flavonoid moiety) with one of the most important enzymes in the animal metabolism, the NKA.

Bioavailability of most polyphenolic compounds naturally found in silymarin is limited by their lower solubility in water, only SCH and SD exhibit substantially higher solubility in aqueous environment. In our experiments, we observed that only SCH inhibited the NKA with IC_50_ of 110 ± 40 μM, and similar concentrations can hardly be reached within the living organism. On the other hand, two of the 2,3-dehydroderivatives, DHSCH and DHSD, inhibited NKA substantially more efficiently with IC_50_ of 38 ± 8 μM and 36 ± 14 μM, respectively. Interestingly, none of the flavonolignans exhibited influence on the known K^+^/ouabain antagonism (Müller-Ehmsen et al., 2001). Moreover, in contrast to ouabain, they did not alter the K^+^-dependence of NKA activity, suggesting that the flavonolignans have different mode of inhibition than ouabain.

Interaction of small molecules with large biomolecules is one of the key issues in structural biology, and recently, it has gained benefits from the development in computational techniques. The classical key-and-lock concept assumes that small molecules can specifically interact with enzymes when their geometries fit each other. The induced-fit concept that was proposed later, has not that strict requirements on the geometries of the interaction partners, and it assumes that after the first contact, the enzyme can rearrange its conformation to fit the shape of the small ligand (Koshland, 1995).

Our calculations revealed that all flavonolignans (including the dehydro- derivatives) can adopt several stable conformations. Their stabilizing energies calculated in the gas phase appeared very similar, suggesting that they are all present in solution in roughly equimolar mixtures. Although differences between flavonolignans conformers may play no important role in, e.g., their redox properties, the precise geometry is crucial in the interaction with enzymes, and some conformers can be tightly bound to the enzyme, while the others can be inactive.

However, the situation is probably more complex in our case. The fitting of the inhibition curves revealed the Hill coefficient different from 1 for all SCH, DHSCH, and DHSD, indicating the presence of multiple binding sites. Indeed, molecular docking revealed one extracellular binding site and four other sites in the cytoplasmic segment of the protein, indicating multiple possibilities of how the flavonolignans could inhibit the enzyme. The main enzyme function is translocation of cations through the plasma membrane. The cations are transiently bound to their binding sites formed by residues TM4, TM5, TM6, and TM8 in the transmembrane domain (Morth et al., 2007), while the extracellular- and cytoplasmic pathways to these binding sites alternatively open and close. Binding of flavonolignans to the extracellular binding site or to the C-terminal binding site can efficiently block these pathways, and thus, can stop the cation transport. The opening and closing of the extracellular- and cytoplasmic gates is accompanied by the change in the position of cytoplasmic domains, as a consequence of ATP-binding and hydrolysis (Kubala, 2006). Localization of the flavonolignan on the interfaces between N- and P- or N- and A-domains can hinder the interactions between cytoplasmic domains, and thus, inhibit the enzyme allosterically. All conformers of all SCH, DHSCH, and DHSD were able to bind to at least to one binding pose, but by far the most occupied site was the one at the interface of N- and P-domains near the phosphorylation site. It can be occupied by almost all conformers, it is present in both opened and closed enzyme conformations, and we experimentally verified binding of all SCH, DHSCH, and DHSD to the large cytoplasmic segment connecting TM4 and TM5 (C45) using fluorometry.

The C45 constitutes approx. 40% of the enzyme mass and forms the cytoplasmic domains N and P. It can be overexpressed without the rest of the enzyme in high quantities in *E. coli* (Grycova et al., 2009). So far, all the experiments indicate that it retains its structure, ability to bind ATP and TNP-ATP (Kubala et al., 2003a,b, 2004), and dynamic properties (Grycova et al., 2009; Kubala et al., 2009) as when it is a part of the entire enzyme. Its solubility greatly facilitates all subsequent experiments and this model system was successfully used for closer localization of binding sites of numerous other small molecules on the cytoplasmic part of NKA (Huličiak et al., 2012; Havlíková et al., 2013). Also in the case of SCH, DHSCH and DHSD, the spectroscopic experiments unambiguously confirmed that these molecules interact with C45. Moreover, in the case of DHSCH and DHSD, we could observe two peaks in the emission spectra of the protein-bound forms. It reveals that there are two binding modes of these species to the C45 and it is in line with predictions from molecular docking.

In conclusion, flavonolignans are proposed to be a novel class of NKA inhibitors, and particularly the 2,3-dehydroderivatives DHSCH and DHSD seem to be very promising agents. The flavonolignans are distinct from the cardiac glycosides that are currently used in NKA treatment. Because their binding sites are different, the mechanism of inhibition is different as well as the range of active concentrations, one can expect that these new NKA inhibitors would exhibit also a different biomedical actions than cardiac glycosides. Currently, the major problem seems to be the low specificity in interaction with biomolecules, which is probably partially related to significant number of stable flavonolignan conformers. Syntheses of more than 100 flavonolignan derivatives have been described (Biedermann et al., 2014), and further screening could identify a derivative that would be useful at lower concentrations and with higher specificity.

## Author contributions

MK designed the study, prepared the manuscript, performed the absorption- and fluorescence spectroscopy experiments. PČ performed the molecular docking computations, contributed to the interpretation of data. JG isolated NKA and performed the ATPase activity measurements. MB performed the conformational analysis and prepared the flavonolignan molecules for docking. TŠ expressed and purified the C45 protein for spectroscopic analyses. PT participated to the conformational analysis and manuscript preparation. DB isolated, prepared, purified and supplied the compounds studied.

## Funding

This work was supported by the grant LO1024 from the National Program of Sustainability I, by scholarship for MB from the French Embassy in the Czech Republic, and by grants 15-03037S and P208/12/G016 from the Czech Science Foundation.

### Conflict of interest statement

The authors declare that the research was conducted in the absence of any commercial or financial relationships that could be construed as a potential conflict of interest.
